# Impact of Structured Reporting of Lower Extremity CT Angiography on Report Quality and Workflow Efficiency

**DOI:** 10.3390/diagnostics14171968

**Published:** 2024-09-06

**Authors:** Claudius Melzig, Victoria Mayer, Martin Moll, Omar Naas, Sibylle Hartmann, Thuy Duong Do, Hans-Ulrich Kauczor, Fabian Rengier

**Affiliations:** 1 Clinic for Diagnostic and Interventional Radiology, Heidelberg University Hospital, Im Neuenheimer Feld 420, 69120 Heidelberg, Germany; 2 Department of Nuclear Medicine, Heidelberg University Hospital, Im Neuenheimer Feld 400, 69120 Heidelberg, Germany

**Keywords:** structured reporting, report quality, computed tomography angiography, peripheral arterial disease, lower extremity CT angiography

## Abstract

We assessed the effects of structured reporting (SR) of lower extremity CT angiography (CTA) on report quality and workflow efficiency compared with conventional reports (CR). Surveys were conducted at an academic radiology department before and after the introduction of an SR template. Participants (*n* = 39, 21) rated report quality and report creation effort (1: very dissatisfied/low to 10: very satisfied/high) and whether SR represents an improvement over CR (1: completely disagree to 5: completely agree). Four residents and two supervising radiologists created both CR and SR of 40 CTA examinations. Report creation time was measured and the factual accuracy of residents’ reports was judged. Report completeness (median 8.0 vs. 7.0, *p* = 0.016) and clinical usefulness (7.0 vs. 4.0, *p* = 0.029) were rated higher for SR. Supervising radiologists found report clarity improved by SR (8.0 vs. 4.5, *p* = 0.029). Report creation effort was unchanged (7.0 vs. 6.0, *p* > 0.05). SR was considered an improvement over CR (median 4.0, IQR,3.0–5.0). Report supervision was shortened by SR (6.2 ± 2.0 min vs. 10.6 ± 3.5 min, *p* < 0.001) but total time for report creation remained unchanged (36.6 ± 12.8 min vs. 36.4 ± 11.0 min, *p* > 0.05). Factual accuracy of residents’ SR was deemed higher (8.0/9.5 vs. 7.0/7.0, *p* = 0.006/ < 0.001). In conclusion, SR has the potential to improve report quality and workflow efficiency for lower extremity CTA.

## 1. Introduction

Structured reporting (SR) is being increasingly used in clinical routine in radiology departments [1] and usually encompasses an electronic report template with predefined headings and subheadings, and potentially point-and-click-based items [2]. Several studies have investigated the effects of structured reporting and suggested improvements in report quality, factual accuracy and satisfaction of referring physicians compared with conventional free-text reports (CR) [3,4,5,6,7]. Furthermore, SR may improve the extraction of relevant information for both, clinical and research purposes [8]. SR has also been reported to be beneficial for educational purposes by guiding the assessment of relevant structures and features [9,10]. This may be even more valuable in a training environment such as academic institutions where typically a preliminary report is created by a resident and later on supervised by a board-certified radiologist [10,11,12]. However, published results on the effects of SR are heterogenous with some studies indicating, for example, no improvement in report accuracy [13,14]. Data on the effects of SR on the radiology workflow are more scarce and some publications have suggested no clear benefit [15] or even a negative impact on efficiency [14,16,17]. Overall, the current level of evidence on the benefits and drawbacks of structured reporting is limited [18] and hampered by an unclear definition of SR in the literature [2].

Computed tomography angiography (CTA) of the lower extremities is a well-established means of characterization of peripheral arterial disease and treatment planning. Clear and concise reporting of these examinations is necessary in order to properly inform clinical decision-making. This may be challenging, especially for the inexperienced, due to the frequently complex nature of the findings and requires a decent knowledge of the relevant anatomy [19]. To our knowledge, only one study to date has been published on the effects of structured reporting of lower extremity CTA on report quality in patients with known or suspected peripheral arterial disease [5]. This study found superior clarity, completeness, clinical relevance, and usefulness of structured reports compared to conventional reports, as judged by referring physicians. Mean report creation times of 10.5 min for SR and 9.0 min for CR were reported for only a small subset of reports [5]. However, the study did not investigate factual accuracy, workflow efficiency and radiologists’ satisfaction with either report form in detail. Therefore, this study aims to investigate whether a highly itemized structured report template for lower extremity CTA examinations significantly alters report quality, workflow efficiency and/or radiologists’ satisfaction compared with conventional free-text reports.

## 2. Materials and Methods

### 2.1. Structured Reporting of Lower Extremity CTA

**Figure 1:**
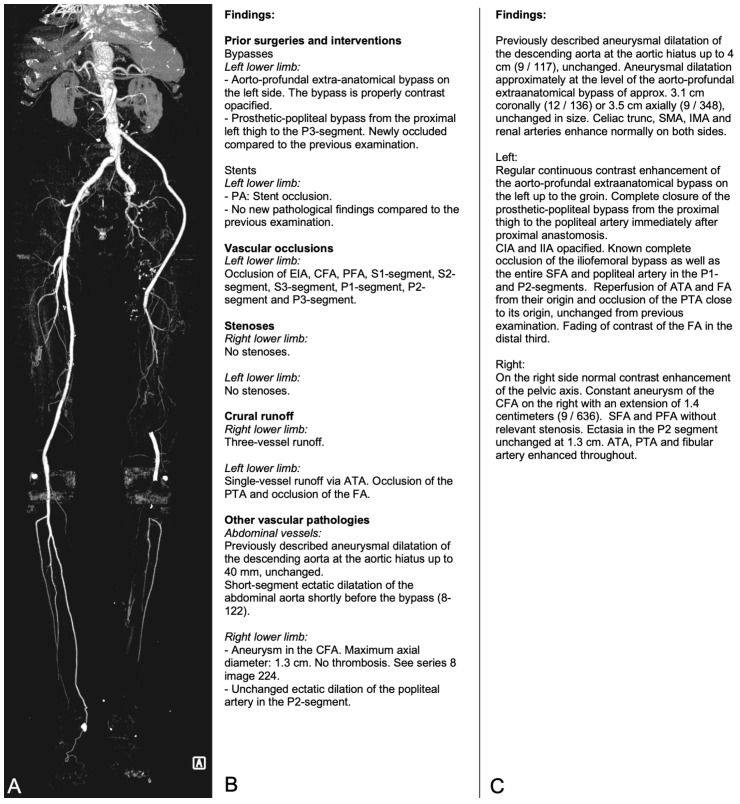
Example of lower extremity CTA examination and corresponding structured and conventional reports. A maximum intensity projection (view from anterior, indicated by “A” in the figure) of a lower extremity CTA in a 68-year-old patient, presenting with subacute borderline left lower limb ischemia (**A**). Corresponding vascular findings were created using the structured report template (**B**) or conventional free-text (**C**). ATA: anterior tibial artery, CFA: common femoral artery, EIA: external iliac artery, FA: fibular artery, IMA: inferior mesenteric artery, PA: popliteal artery, P1–3: popliteal artery segments 1–3, PFA: profunda femoris artery, PTA: posterior tibial artery, SFA: superficial femoral artery, S1–3: superficial femoral artery segments 1–3, SMA: superior mesenteric artery.

Structured reports were created using a commercially available, browser-based structured reporting software (Smart Reporting, Munich, Germany) allowing for point-and-click report creation as well as inclusion of free-text sections. The reporting template used in this study was created in-house and consisted of sections on general information, such as patient history, premedication and image quality, vascular and extravascular findings and impression. The vascular findings were further subdivided into prior vascular interventions, occlusions/stenoses and other vessel-related pathologies. Vascular findings were highly itemized to minimize the need for free-text input, and, whenever possible, a schematic was provided to allow point-and-click selection of the anatomical location of vascular findings. An example of a lower extremity CTA examination with corresponding SR and CR vascular findings is shown in Figure 1. The template was created in a multi-stage iterative process by two experienced vascular radiologists (CM and FR) and modified based on other radiologists’ feedback before introduction into their daily routine. Department-wide introduction of the template was accompanied by an introductory presentation on template functionality and guided use of the template in clinical routine by the template creators for the first four weeks.

### 2.2. Surveys

Two anonymous surveys were conducted in our radiology department, which also covers interventional radiology, within a 1-year interval using a web-based service (Google Forms, Google LLC., Mountain View, CA, USA). The links to the survey forms were distributed department-wide via email. The first survey took place 6 months before the introduction of the SR template for lower extremity CTA and the second survey was conducted 6 months thereafter. Both surveys were structured equally with an initial section asking for general information on the participants and a second section consisting of questions on report quality and the reporting process. Participants were asked to rate their overall satisfaction with the respective report form (CR in the first and SR in the second survey) as well as report completeness, clarity and clinical relevance on a 10-point scale from 1 (very dissatisfied) to 10 (very satisfied). Additionally, respondents rated the subjective effort required to create a CTA report in the respective form on a scale from 1 (very low) to 10 (very high). In the second survey, participants were asked whether they had already used the SR template for lower extremity CTA and further responses were only collected from those who stated to have used the template. Additionally, participants in the second survey were also asked whether structured reporting represents an improvement over conventional reports for lower extremity CTA on a scale from 1 (completely disagree) to 5 (completely agree). Finally, free-text responses were collected in the second survey on perceived advantages and disadvantages of SR for lower extremity CTA and recommendations for potential improvements of SR for CTA. The surveys were pre-tested with two board-certified radiologists to ensure comprehensibility and minor changes to presentation and spelling were carried out accordingly.

### 2.3. Report Creation Time and Report Accuracy

To systematically compare the time required for the creation of CR and SR of lower extremity CTA an analysis independent of the department-wide surveys was conducted. A focus group of 4 residents (ON, MM, SS and VM) with 3 to 6 years of radiology training and two supervisors, both board-certified radiologists (CM and TD) with 8 and 10 years of experience in radiology, was assembled. A power analysis for a paired Student’s *t*-test was conducted to determine the number of required cases for the analysis using the following assumptions: minimum relevant difference in mean reporting time 5 min (300 s), estimated standard deviation of reporting time based on the literature and clinical experience 10 min (600 s), power 0.08, significance level 0.05, resulting in a minimum number of 34 cases required. Correspondingly, 40 consecutive lower extremity CTA examinations were identified. The 4 residents were then randomly assigned to each create CRs for 10 of the examinations and SRs for another 10 examinations, resulting in a new CR and SR for each of the 40 examinations and 80 reports in total. These reports were then randomly assigned to the 2 supervisors so that each supervisor reviewed and edited either a CR or an SR for all of the examinations. The time required for the creation of preliminary reports by residents as well as review and revision of these reports by supervisors was measured. The time was taken from the beginning of the image review to the finalization of the report. Additionally, the supervisors rated their satisfaction with the factual accuracy of each respective preliminary report on a scale from 1 (very dissatisfied) to 10 (very satisfied). To minimize variability, all examinations were prepared with identical hangings in the picture archiving and communication system (PACS) and all images had to be loaded to the workstation prior to image review. Images were reviewed via Centricity PACS Radiology RA1000 (GE Healthcare, Chicago, IL, USA) same as in clinical routine in our institution. Readers were blinded to the original reports created in the clinical routine.

### 2.4. Template Adoption Rate

To monitor usage of the structured report template, all reports of lower extremity CTA examinations during the 12 months after the introduction of the template were retrospectively reviewed and categorized as CR or SR based on the presence of an identifying report header of the SR template. For each quarter of the year after the template introduction, the ratio of SR to all lower extremity CTAs was calculated. Employees of the department were not informed about the monitoring process and, except during the introductory first 4 weeks, template adoption was not actively encouraged.

### 2.5. Statistical Analysis

Shapiro–Wilk test and Q–Q plots were employed to test for normal distribution. Normally distributed continuous data were expressed as mean +/− standard deviation, non-normally distributed data were expressed as median and interquartile range (IQR) and categorical data were expressed as numbers and percentages. Group comparison between non-normally distributed and ordinal data was conducted using Wilcoxon rank-sum tests and normally distributed data were compared using a two-sided Student’s *t*-test, where appropriate. Categorical data were compared using the Chi-squared test of independence. Multivariable linear regression analysis was used to estimate the influence of potential confounders on perceived report factual accuracy. Free-text comments for each question were retrospectively coded into categories derived from the comments themselves by one investigator (CM) in a grounded theory qualitative analysis approach [20]. The occurrence rate of each comment category was calculated in relation to the number of respondents. *p* values < 0.05 were considered statistically significant. Statistical analyses were conducted using R Version 4.2.2 (R Foundation for Statistical Computing, Vienna, Austria).

## 3. Results

### 3.1. Characterization of Survey Participants

Characteristics of participants of the department-wide surveys are summarized in Table 1. In total, 39 radiologists participated in the survey on conventional reports for lower extremity CTA and 21 participated in the survey on SR. The majority of the participants in both surveys were residents, 27/39 (69.23%) in the CR survey and 14/21 (66.67%) in the SR report survey (*p* > 0.05). Reported years of experience as a radiologist did not differ significantly between CR and SR survey participants (median 4.0 years (IQR, 3.0–7.5) in CR and 4.0 years (IQR, 3.0–6.8) in the SR survey, *p* > 0.05). There was also no statistically significant difference in the self-reported experience level on the reporting of lower extremity CTA examinations, with the majority of participants judging themselves as being “competent” in the interpretation of these examinations (19/3, 48.72%, in the CR survey and 8/21, 38.10%, in the SR survey, *p* > 0.05).

### 3.2. Overall Satisfaction with CTA Reports

**Figure 2:**
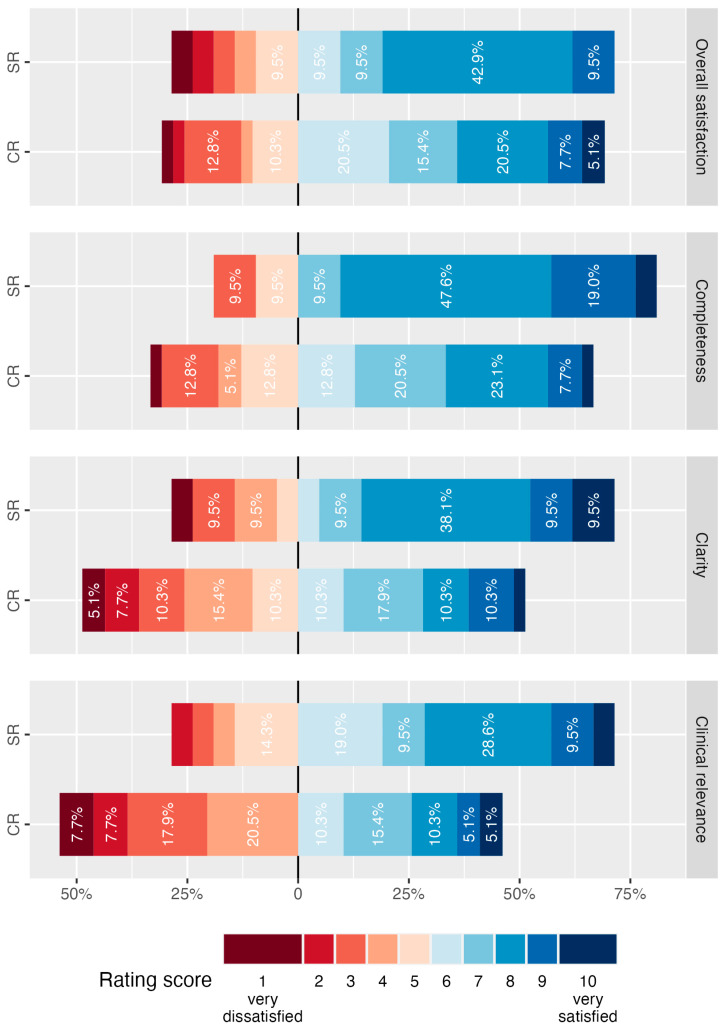
Results of department-wide survey on report quality of lower extremity CTA. Participants were asked to express their satisfaction with various aspects of the quality of structured reports (SR) and conventional reports (CR) for lower extremity CTA on a scale from 1 (very dissatisfied) to 10 (very satisfied). Responses of all survey participants are summarized in diverging stacked bar charts and percentages for each rating per report form and quality aspect are illustrated.

**Figure 3:**
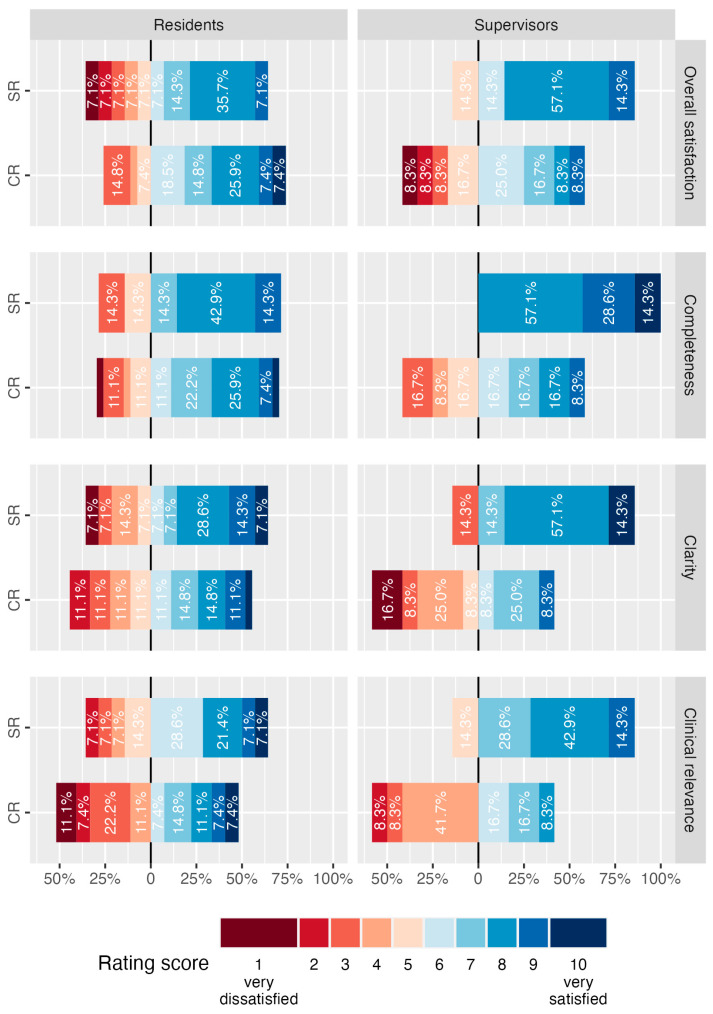
Results of department-wide survey on report quality of lower extremity CT angiographies disaggregated by level of education. Participants were asked to express their satisfaction with various aspects of the quality of structured reports (SR) and conventional reports (CR) for lower extremity CTA on a scale from 1 (very dissatisfied) to 10 (very satisfied). Responses of survey participants, disaggregated by their level of education in radiology are summarized in diverging stacked bar charts and percentages for each rating per level, report form and quality aspect are illustrated.

Survey results on report quality are summarized in Figure 2 and Figure 3. Participants in the department-wide survey were asked to judge their overall satisfaction with each report form for lower extremity CTA on a scale from 1 (very dissatisfied) to 10 (very satisfied). Overall satisfaction was 8.0 (IQR, 5.0–8.0) for SR and 6.0 (IQR, 5.0–8.0) for CR (*p* > 0.05). Median ratings of residents were 7.0 (IQR, 4.2–8.0) for SR and 7.0 (IQR, 5.5–8.0) for CR (*p* > 0.05). Median ratings of supervising radiologists were 8.0 (IQR, 7.0–8.0) for SR and 6.0 (IQR, 4.5–7.0) for CR (*p* > 0.05).

### 3.3. Satisfaction with Report Completeness

On a scale from 1 (very dissatisfied) to 10 (very satisfied), report completeness was rated significantly higher for SR with a median of 8.0 (IQR, 7.0–8.0) compared with a median of 7.0 (IQR, 5.0–8.0) for CR (*p* = 0.016). Supervising radiologists rated SR completeness with 8.0 (IQR, 8.0–9.0) compared to 6.0 (IQR, 4.8–7.2) for CR (*p* = 0.005). Median ratings of residents did not differ significantly with 8.0 (IQR, 5.5–8.0) for SR vs. 7.0 (IQR, 5.0–8.0) for CR (*p* > 0.05).

### 3.4. Satisfaction with Report Clarity

On a scale from 1 (very dissatisfied) to 10 (very satisfied), survey participants’ median rating for clarity of reports for lower extremity CTA was 8.0 (IQR, 5.0–8.0) for SR and 6.0 (IQR, 4.0–7.0) for CR (*p* = 0.050). Supervising radiologists’ satisfaction with clarity was significantly improved by SR with median ratings of 8.0 (IQR, 7.5–8.0) compared with 4.5 (IQR, 3.8–7.0) for CR (*p* = 0.029). No statistically significant difference was detected in the group of residents with median ratings of 7.5 (IQR, 4.2–8.0) for SR vs. 6.0 (IQR, 4.0–8.0) for CR (*p* > 0.05).

### 3.5. Estimation of Clinical Relevance of Reports

Clinical relevance of lower extremity CTA reports was judged by survey participants on the same scale from 1 (very low) to 10 (very high) for each report form. Overall ratings were significantly higher for SR (median 7.0, IQR, 5.0–8.0) compared with CR (4.0, IQR, 3.0–7.0, *p* = 0.029). However, the difference in ratings was not statistically significant for the group of residents (6.0, IQR, 5.0–8.0 for SR versus 4.0, IQR, 3.0–7.5 for CR, *p* > 0.05), whereas supervising radiologists judged SR to be of significantly higher clinical relevance (8.0, IQR, 7.0–8.0 for SR versus 4.0, IQR, 4.0–6.2 for CR, *p* = 0.008).

### 3.6. Perceived Effort for Report Creation

**Figure 4:**
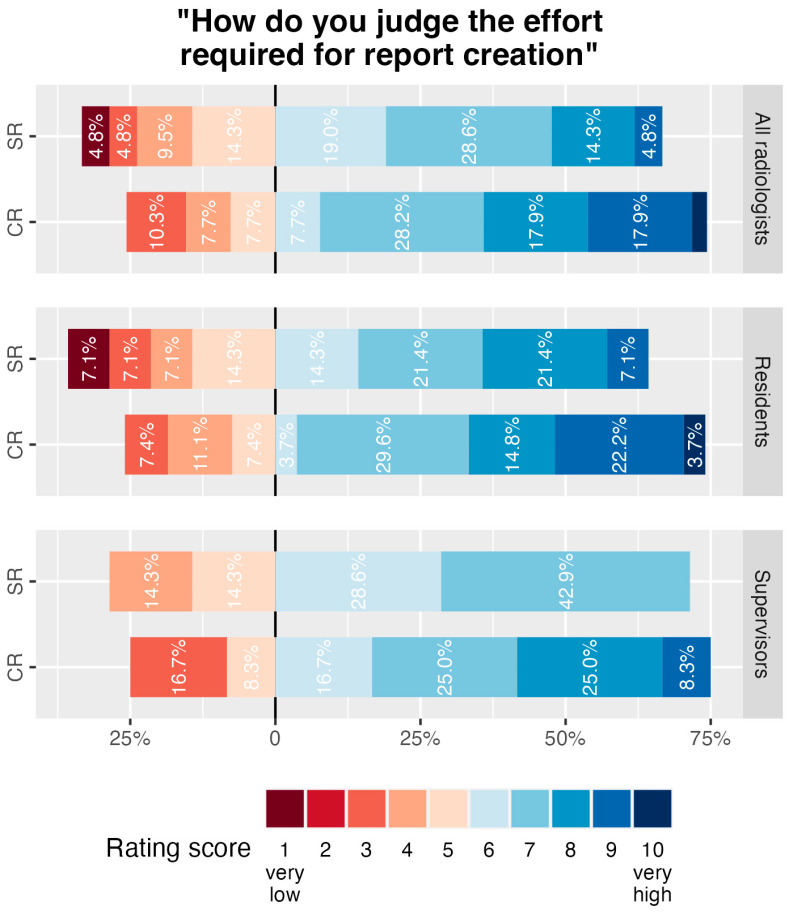
Results of department-wide survey on subjective effort required for report creation for lower extremity CT angiographies. Participants’ evaluation of subjective effort required to create structured reports (SR) or conventional reports (CR) for lower extremity CTA on a scale from 1 (very low) to 10 (very high). Responses of all survey participants ((top) row) as well as disaggregated by level of education in radiology, are summarized in diverging stacked bar charts and percentages for each rating per report form and education level are given.

Survey results on the subjective effort required for the creation of reports as rated by participants of the department-wide survey are summarised in Figure 4. Subjective effort was rated on a scale from 1 (very low) to 10 (very high). No statistically significant difference was identified for the median responses of all participants (7.0, IQR, 5.5–8.0, for CR and 6.0, IQR, 5.0–7.0, for SR, *p* > 0.05). Residents’ median ratings were 7.0 (IQR, 5.5–8.5) for CR and 6.5 (IQR, 5.0–7.8, *p* > 0.05) for SR and supervising radiologists’ median responses were 7.0 (IQR, 5.8–8.0) for CR and 6.0 (IQR, 5.5–7.0, *p* > 0.05) for SR.

### 3.7. Improvement over Conventional Reports

**Figure 5:**
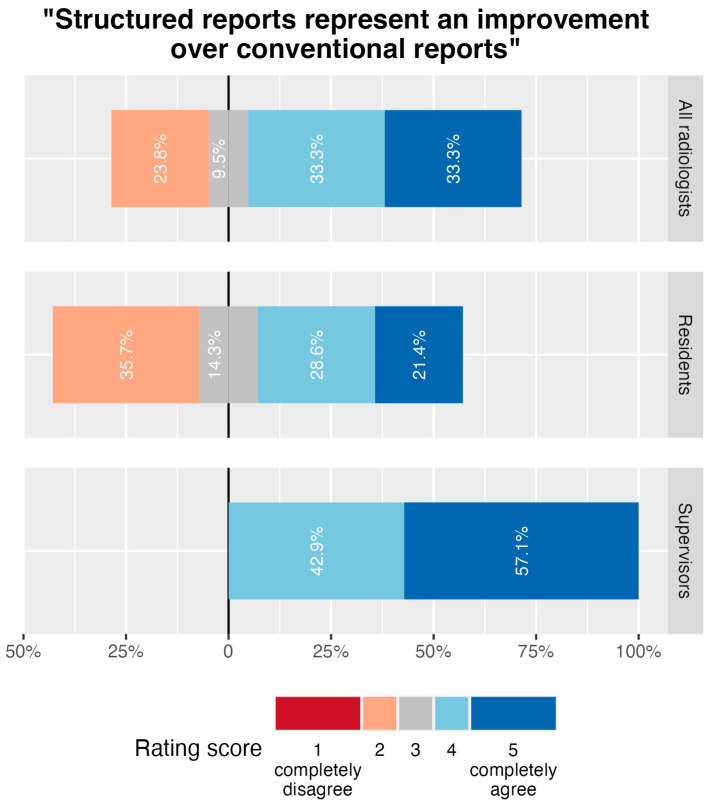
Results of department-wide survey on the statement “Structured reports represent an improvement over conventional reports”. Participants were asked to judge the statement “structured reports represent an improvement over conventional reports” on a scale from 1 (completely disagree) to 5 (completely agree). Responses of all survey participants ((top) row) as well as disaggregated by level of education in radiology, are summarized in diverging stacked bar charts and percentages for each rating are given.

The statement “structured reports represent an improvement over conventional reports for lower extremity CTA examinations” was rated on a scale from 1 (completely disagree) to 5 (completely agree). The median response of all participants in the department-wide survey was 4.0 (IQR, 3.0–5.0), with significantly higher agreement in the group of supervising radiologists (median 5.0, IQR, 4.0–5.0) compared with the group of residents (median 3.5, IQR, 2.0–4.0; *p* = 0.032; Figure 5).

### 3.8. Analysis of Comments on Advantages, Disadvantages and Potential Improvements of SR

Categorized free-text comments on advantages, disadvantages and potential improvements of structured reports for lower extremity CTA according to survey participants are summarised in Table 2. Improved standardization, improved clarity and completeness were the most frequently mentioned advantages of structured reports (60%, 50% and 40% of commentators, respectively). The most frequently listed disadvantage was an increase in perceived effort for report creation (80% of respondents). Half of the commentators listed technical improvements as an area of potential improvement for SR. In total, 25% of the respondents wished for increased flexibility of the template and also 25% wished for an altered structure of the template.

### 3.9. Report Creation Time

Average times for creation of preliminary reports by residents were 30.4 ± 12.1 min (range 5.2–60 min) for SR and 25.9 ± 9.6 min (range 9.9–50.3 min) for CR. The difference was not statistically significant (*p* > 0.05). Average times for report review and editing by supervising radiologists were significantly shorter for SR (6.2 ± 2.0 min, range 2.8–10.1 min) compared with CR (10.6 ± 3.5 min, range 2.7–19.7 min, *p* < 0.001). However, the average combined time invested by both, resident and supervisor, per report did not differ significantly between SR (36.6 ± 12.8 min, range 8.2–64.9 min) and CR (36.4 ± 11.0 min, range 12.5–60.0 min, *p* > 0.05).

### 3.10. Factual Accuracy of Preliminary Reports

Median ratings by supervisor A on the factual accuracy of preliminary reports were 8.0 (IQR, 8.0–9.0) for SR and 7.0 (IQR, 5.8–8.2, *p* = 0.006) for CR, whereas supervisor B’s median ratings were 9.5 (IQR, 8.0–10.0) for SR and 7.0 (IQR, 6.0–8.0, *p* < 0.001) for CR. A multivariable linear regression analysis including report form, supervisor and the interaction term between the two as covariates was conducted to assess the potentially significant influence of the respective supervisor on the factual accuracy ratings (Table S1). No significant influence of the respective supervisor on the factual accuracy rating was detected, whereas the report form significantly contributed to the explanation of the factual accuracy ratings in the model.

### 3.11. Adoption Rate of Structured Report Template

The usage of the structured report template for lower extremity CTA examinations was investigated for the 1-year period after introduction into the clinical routine. The ratio of structured reports in relation to all lower extremity CTA examinations performed was 52.0% (39/75) in the first quarter, 59.2% (45/76) in the second quarter, 63.1% (41/65) in the third quarter and 66.2% (51/77) in the fourth quarter of that year.

## 4. Discussion

We assessed report quality and workflow efficiency of structured reporting for lower extremity CTA compared with conventional free-text reports. In a department-wide survey, radiologists’ satisfaction with report completeness and clinical relevance was significantly higher for structured reports compared with conventional reports and supervising radiologists also judged report clarity to be significantly improved by structured reporting. Structured reports were considered to be an improvement over conventional reports, especially among the group of supervising radiologists. Structured reports created by residents were judged to be significantly more factually accurate compared with conventional reports, as assessed by two experienced supervising radiologists. No statistically significant difference in perceived effort required for report creation was found between structured and conventional reporting. In a systematic analysis, the total time required for report creation did not differ significantly between structured and conventional reports. However, the time required for review and editing by supervising radiologists was significantly shorter for structured reports compared with conventional reports.

To our knowledge, only one prior study to date investigated structured reporting for lower extremity CTA [5]. Sabel and colleagues assessed the quality of structured and conventional reports as evaluated by referring vascular surgeons and vascular medicine specialists. Similar to our results, they found greater satisfaction with report completeness, clarity, clinical relevance and usefulness for SR compared with CR [5]. In addition to that, the factual accuracy of residents’ preliminary reports was assessed in our study and judged to be significantly higher for SR. Varying results have been published on report accuracy and key findings reporting in SR versus CR for various imaging examinations. While some studies highlighted lower rates of missing key findings in SR versus CR [6,21,22], others have shown no improvement in factual accuracy [13,14]. However, as highlighted in a recent systematic review, study outcomes on structured reporting may be highly case-specific [18].

Sabel and colleagues also analyzed report creation time for lower extremity CTA in a small set of five randomly chosen cases and reported mean times of 10.5 min (range 6.0 to 14.0 min) for SR and 9.0 min (range 6.0–12.0 min) for CR [5]. However, a detailed analysis of the time and effort spent by residents and supervisors for report creation for lower extremity CTA has not been published to date. While the most frequent free-text comment on the disadvantages of SR in our study was a perceived increase in effort, we found no significant difference in the ratings on perceived effort and overall time spent for report creation. On the other hand, we did find a significantly reduced time required for report supervision by board-certified radiologists from 10.6 ± 3.5 min for CR to 6.2 ± 2.0 min for each report. This improvement in efficiency especially for the group of supervising radiologists with usually higher workloads and salaries could potentially translate into a relevant reduction in workload and cost for report creation using SR. Several previous studies have demonstrated improved report turnaround times for other examinations [23,24,25] and a recent study has demonstrated a mean reduction in report turnaround time of 35.0 min for final reports along with a significant reduction in proofreading workload in clinical routine in a neuroradiology department [26]. However, other factors such as costs associated with potential software acquisition, report template creation and implementation as well as potential future changes to the template have to be taken into consideration.

Over the period of 1 year after the introduction of the SR template, we observed a continuous increase in the adoption rate of the template from 52.0% up to 66.2% of all lower extremity CTA examinations in the fourth quarter of that period. The rate may appear low compared with what has been published for other structured report templates, for example, usage rates of up to 100% have been published in an emergency radiology setting [27] or 81% structured template adoption over a variety of modalities and examinations in a study by Hanna et al. [15]. However, the constant increase in usage of our template over time without any additional measures may indicate a learning process leading to increased use of the report template and therefore the potential maximum adoption rate without interference may not have been reached during the observation period. This is supported by free-text comments in the survey in which 30% of the participants cited the associated learning curve as a disadvantage of the SR template. Usage of the template in daily routine is therefore not only dependent on the template itself but also on the processes associated with its introduction [28].

A common drawback of studies on structured reporting is a potential dependence of the results on the specific technical implementation and template used in each study, as has recently been pointed out in a systematic review [18]. As identified in the analysis of free text comments in our study, technical aspects and the structure of the report as defined by the template influenced the perceived benefits and drawbacks of the structured report. Therefore, the generalizability of our results is limited. However, as outlined above, our results point in a similar direction to what has been published for this type of examination and structured reporting in general. Similarly, the comparably small number of participating supervising radiologists in our study is a limitation and the results therefore require future verification in a larger, ideally multi-center study. Another potential limitation of our study is the analysis of report creation time in a focus group rather than the analysis of report turnaround time in a daily routine. This approach was chosen in order to minimize the influence on reporting time by other factors, such as phone calls, conference preparations, etc., and more precisely measure the time actually spent by the two readers on report creation. Whereas the absolute time may not be directly translatable to the daily routine, we would expect the direction of change to be the same. This may be supported by the results of our department-wide survey which showed that structured reporting was considered an improvement, especially among supervising radiologists, and the continuous increase in template usage in daily routine over the observation period.

## 5. Conclusions

Structured reporting improved report quality and reduced the time needed for report supervision compared with conventional reports for lower extremity CTA. While an increase in effort for report creation was the most common free-text comment on the disadvantages of SR, we detected no significant difference in perceived effort ratings, and structured reports were overall considered an improvement over conventional reports, especially among supervising radiologists. These results are based on a single-center experience and therefore warrant further investigation. However, they highlight and underline the potential benefits of structured reporting in the setting of lower extremity CTA examinations.

## Figures and Tables

**Table 1:** Characteristics of survey participants.

	Participants CR Survey	Participants SR Survey	*p*-Value
N	39	21	
Gender			
Male	23 (59.0%)	14 (66.6%)	0.38
Female	10 (25.6%)	6 (28.6%)	
Other/undisclosed	6 (15.4%)	1 (4.8%)	
Education level			
Resident	27 (69.2%)	14 (66.6%)	1.00
Supervising radiologist	12 (30.8%)	7 (33.3%)	
Years of experience in radiology	4.0 (IQR, 3.0–7.5)	4.0 (IQR, 3.0–6.8)	0.94
Experience with lower extremity angiography			
Novice	5 (12.8%)	1 (4.8%)	0.66
Advanced	7 (18.0%)	5 (23.8%)	
Competent	19 (48.7%)	8 (38.1%)	
Experienced	6 (15.4%)	5 (23.8%)	
Expert	2 (5.1%)	2 (9.5%)	

Values are presented as the number of participants with percentage in parentheses, or median with interquartile range (IQR) in parentheses, as appropriate according to Shapiro–Wilk test of distribution. CR: conventional report, SR: structured report.

**Table 2:** Analysis of participants’ comments on advantages, disadvantages and potential improvements of structured reporting for lower extremity CT angiography examinations.

Category	Example Comments	Absolute Frequency	Relative Frequency
Advantages of structured reporting for lower extremity CT angiography examinations			
Standardization	“Unified terminology” “Improved comparability”	6	60.0%
Clarity	“Improved clarity”	5	50.0%
Completeness	“One will not forget to include items in the report”	4	40.0%
Usefulness	“Good for standard situations”	1	10.0%
Education	“Template serves as an instruction to report creation (education)”	1	10.0%
Orthography	“No misspellings due to itemized report creation”	1	10.0%
Disadvantages of structured reporting for lower extremity CT angiography examinations			
Effort	“Report creation is prolonged”	8	80.0%
Clarity	“Reduced clarity in complex situations”	3	30.0%
Learning curve	“Increased effort to become acquainted with the report creation process”	3	30.0%
Technical limitations	“Technical limitations to the automatic assembly of the impression section based on the findings”	1	10.0%
Potential improvements to structured reporting for lower extremity CT angiography examinations			
Technical improvements	“Graphical representation of the pathologies in the report is desirable” “Improved automatic assembly of impression section based on findings/stenoses in the report”	4	50.0%
Increased flexibility	“Include more detailed selection for what to include automatically in the impression section of the report”	2	25.0%
Structure	“The report template is currently structured according to the type of pathology (stenosis, occlusion etc. I would rather prefer a structure based on the anatomical location of the pathologies”	2	25.0%
Instruction	“Systematic introduction of the template in the beginning”	1	12.5%

## Data Availability

The datasets presented in this article are not readily available because the data are part of an ongoing study. Requests to access the datasets should be directed to the corresponding author.

## Supplementary Materials

The following supporting information can be downloaded at: https://www.mdpi.com/article/10.3390/diagnostics14171968/s1, Table S1: Regression analysis of factual accuracy of preliminary reports as evaluated by two supervising radiologists.
