# Supplementary material for: Impact of Structured Reporting of Lower Extremity CT Angiography on Report Quality and Workflow Efficiency
Source: Diagnostics (Basel). 2024 Sep 6;14(17):1968. doi: 10.3390/diagnostics14171968 (PMC11394164; doi:10.3390/diagnostics14171968)
Supplement: Supplementary file 1 [file diagnostics-14-01968-s001.zip › diagnostics-3153643-supplementary.pdf]

Table S1

Regression analysis of factual accuracy of preliminary reports as evaluated by two supervising radiologists.

	Variables	Estimate [95%-CI]	Standard error		p-value	Adjusted r²
Model 1	Intercept	6.750 [6.116;7.384]	0.318	--	<0.001	0.304
	Report form (0: CR, 1: SR)	1.600 [-0.596;1.196]	0.450	0.472	<0.001	
	Supervisor (0: supervisor A, 1: supervisor B)	0.300 [0.704;2.496]	0.450	0.089	0.51	
	Report form X Supervisor	0.500 [-0.767;1.767]	0.636	0.148	0.43	
Model 2	Intercept	6.625 [6.078;7.172]	0.275	--	<0.001	0.307
	Report form (0: CR, 1: SR)	1.850 [-0.082;1.182]	0.317	0.546	<0.001	
	Supervisor (0: supervisor A, 1: supervisor B)	0.550 [1.218;2.482]	0.317	0.162	0.09	
	Report form X Supervisor	--	--	--	--	
Model 3	(Intercept)	7.550 [7.017;8.083]	0.268	--	<0.001	0.014
	Report form (0: CR, 1: SR)	--	--	--	--	
	Supervisor (0: supervisor A, 1: supervisor B)	0.550 [-0.204;1.304]	0.379	0.162	0.15	
	Report form X Supervisor	--	--	--	--	
Model 4	Intercept	6.900 [6.448;7.352]	0.227	--	<0.001	0.289
	Report form (0: CR, 1: SR)	1.850 [1.210;2.490]	0.321	0.546	<0.001	
	Supervisor (0: supervisor A, 1: supervisor B)	--	--	--	--	
	Report form X Supervisor	--	--	--	--	

CR: conventional report, SR: structured report, CI: confidence interval, X: interaction term
